# Curcumin suppresses epithelial-to-mesenchymal transition of peritoneal mesothelial cells (HMrSV5) through regulation of transforming growth factor-activated kinase 1 (TAK1)

**DOI:** 10.1186/s11658-019-0157-x

**Published:** 2019-05-22

**Authors:** Jun-Li Zhao, Mei-Zi Guo, Jun-Jun Zhu, Ting Zhang, Dan-Yan Min

**Affiliations:** 1 Department of Nephrology, Shanghai University of Medicine & Health Sciences affiliated Zhoupu Hospital, Pudong New District, Shanghai, 201318 China; 2 Department of Geriatrics, Shanghai University of Medicine & Health Sciences affiliated Zhoupu Hospital, Pudong New District, Shanghai, 201318 China

**Keywords:** Peritoneal fibrosis (PF), Peritoneal dialysis, Epithelial-mesenchymal transition (EMT), Curcumin, TGF-β-activated kinase 1 (TAK1)

## Abstract

**Objective:**

Peritoneal fibrosis remains a serious complication of long-term peritoneal dialysis (PD) leading to peritoneal membrane ultrafiltration failure. Epithelial–mesenchymal transition (EMT) of peritoneal mesothelial cells (PMCs) is a key process of peritoneal fibrosis. Curcumin has been previously shown to inhibit EMT of renal tubular epithelial cells and prevent renal fibrosis. There are only limited reports on inhibition of PMCs-EMT by curcumin. This study aimed to investigate the effect of curcumin on the regulation of EMT and related pathway in PMCs treated with glucose-based PD.

**Methods:**

EMT of human peritoneal mesothelial cells (HMrSV5) was induced with glucose-based peritoneal dialysis solutions (PDS). Cells were divided into a control group, PDS group, and PDS group receiving varied concentrations of curcumin. Cell Counting Kit-8 (CCK-8) assay was used to measure cell viability, and a transwell migration assay was used to verify the capacity of curcumin to inhibit EMT in HMrSV5 cells. Real-time quantitative PCR and western blot were used to detect the expression of genes and proteins associated with the EMT.

**Results:**

High glucose PDS decreased cell viability and increased migratory capacity. Curcumin reversed growth inhibition and migration capability of human peritoneal mesothelial cells (HPMCs). In HMrSV5 cells, high glucose PDS also decreased expression of epithelial markers, and increased expression of mesenchymal markers, a characteristic of EMT. Real-time RT-PCR and western blot revealed that, compared to the 4.25% Dianeal treated cells, curcumin treatment resulted in increased expression of E-cadherin (epithelial marker), and decreased expression of α-SMA (mesenchymal markers) (*P* < 0.05). Furthermore, curcumin reduced mRNA expression of two extracellular matrix protein, collagen I and fibronectin. Curcumin also reduced TGF-β1 mRNA and supernatant TGF-β1 protein content in the PDS-treated HMrSV5 cells (*P* < 0.05). Furthermore, it significantly reduced protein expression of p-TAK1, p-JNK and p-p38 in PDS-treated HMrSV5 cells.

**Conclusions:**

Our results demonstrate that curcumin showed an obvious protective effect on PDS-induced EMT of HMrSV5 cells and suggest implication of the TAK1, p38 and JNK pathway in mediating the effects of curcumin in EMT of MCs.

## Introduction

Peritoneal dialysis (PD) is an effective renal replacement strategy for patients with end-stage renal disease (ESRD). However, continual exposure of the peritoneal membrane (PM) to non-physiological PD solutions, including high concentrations of glucose and lactate, glucose degradation products (GDPs), and low pH [1], may cause acute and chronic inflammation and injury of the PM. In these conditions, the peritoneum undergoes progressive fibrosis, angiogenesis and hyalinizing vasculopathy. These morphological alterations are associated with increased rates of small-solute transport and with ultrafiltration failure (UFF) of the peritoneal membrane, eventually leading to the discontinuation of PD therapy [2, 3]. The mechanism of peritoneal fibrosis is still not fully clear, but it is widely accepted that accumulation of activated myofibroblasts is mainly responsible for this process. Given their nature and the pathological changes during peritoneal impairment, it has been suggested that mesothelial cells through an epithelial-to-mesenchymal transition (EMT) may contribute to the pool of fibroblasts [4].

EMT represents a complex phenomenon of cellular transdifferentiation that converts the epithelial phenotype into a mesenchymal one, which is characterized by disruption of adherent and intercellular tight junctions, adoption of cell polarization and conversely, acquisition of migratory and invasive capacity [5],which allows mesothelial cells (MCs) to invade the submesothelial compact zone and acquire the capacity for synthesis of pro-inflammatory and pro-angiogenic factors, as well as extracellular matrix components. All these events are a physiologic process of peritoneal membrane repair responses provoked by PD but can also promote peritoneal fibrosis in nonphysiologic conditions. High glucose (HG), used as an osmotic agent in most common PD fluids, has been shown to cause fibrosis by upregulating the expression of transforming growth factor-β1 (TGF-β1, [6]) and induction of EMT of peritoneal mesothelium [7, 8]. EMT of mesothelial cells is a reversible process in which epithelial cells transdifferentiate into cells with mesenchymal characteristics, which is widely considered to be a crucial process in fibrosis [7, 9]. Therefore, factors that regulate EMT as an inducer of peritoneal fibrosis have attracted more and more attention.

Currently, no appropriate methods to block peritoneal fibrosis have been approved in clinical practice. Most studies thus far have focused on Chinese medicine materials as an alternative treatment which has been shown to suppress the pro-inflammatory and pro-fibrotic pathway and control PF in several in vivo and/or in vitro studies. Curcumin is a polyphenol isolated from the *Curcuma longa* plant, commonly known as turmeric, which has been routinely used to treat various diseases in China. Modern pharmacological studies suggest that curcumin has many pharmacological effects such as anti-tumor,

anti-inflammatory, anti-fibrosis, and anti-oxidation [10]. Both in vitro and in vivo experiments confirmed that curcumin shows anti-fibrotic effects on liver fibrosis, pulmonary fibrosis and oral submucous fibrosis [11–13]. Recent studies have demonstrated that curcumin has anti-fibrotic effects on renal fibrosis through interfering with TGF-β/Smad signaling pathways, preventing inflammation initiation, inhibiting EMT, and resolving ECM excess deposition in animal models [14]. It is inferred that curcumin has a certain improvement effect on PMCs in the occurrence of EMT and peritoneal fibrosis. However, the protective effects of curcumin against EMT induced by peritoneal dialysis still need to be elucidated. The Smad signaling pathway is widely accepted as a canonical pathway induced by TGF-β1 in the induction of EMT and its reversal. Recently, a large body of evidence has demonstrated that various Smad-independent signaling pathways are involved in the development of EMT and fibrosis [15, 16]. Transforming growth factor-activated kinase-1 (TAK1), a serine/threonine kinase, emerged as a critical upstream signaling molecule in TGF-β-induced Smad-independent signaling pathways. A recent study by Strippoli R [17] showed that TAK1 as a main biochemical mediator mediated EMT and fibrosis in mesothelial cells from human peritoneum. These findings suggest that curcumin may suppress EMT-like changes through the inhibition of TAK1. Here, we used glucose-based PD-induced EMT in mesothelial cells to investigate the role of curcumin in PD-related EMT and to elucidate the exact molecular mechanisms.

## Materials and methods

### Reagents and antibodies

The human peritoneal mesothelial cell line (HMrSV5) was purchased from Shanghai Cell Bank of Chinese Academy of Sciences. Glucose-based peritoneal dialysis solutions (PDS) tested included 1.5% Dianeal, 2.5% Dianeal and 4.25% Dianeal, all from Baxter Medical Co., Ltd. (Guangzhou, China). Standard fetal bovine serum was purchased from Beijing Haiclone. DMEM/F12 medium was purchased from Gibco (USA). Trypsin (0.25%) and EDTA (0.02%) were purchased from Amresco (USA). Curcumin was purchased from Sigma-Aldrich Chemical Corp (St. Louis, MO, USA). A human TGF-β1 ELISA kit was purchased from PeproTech (USA). PrimeScript RT kit (for real time), SYBR Premix Ex Taq II (Tli RNaseH Plus) kit was purchased from Takara (Dalian, China). RNA extraction reagent TRIzol, penicillin and streptomycin were purchased from Invitrogen (Carlsbad, CA, USA). A CCK-8 kit was purchased from Tongren Chemical Co. (Japan). α-SMA rabbit anti-human monoclonal antibody, E-cadherin, phosphorylated TGF-β-activated kinase 1 (p-TAK1), phosphorylated c-Jun N-terminal kinase (p-JNK) and p-p38 mouse anti-human monoclonal antibodies were purchased from Santa Cruz (Santa Cruz, USA).

### Cell culture

Human peritoneal mesothelial cells (HMrSV5) were cultured in DMEM/F12 supplemented with 10% (v/v) heat-inactivated fetal calf serum and 100 U/mL penicillin/streptomycin (Invitrogen). Cells were maintained in a humidified environment containing 5% CO_2_ at 37 °C, and the culture medium was replaced every 2 days. Cells were permitted to attach for 24 h and to grow to 80% confluence. Curcumin was dissolved in DMSO for a stock concentration of 200 mM/L. The maximum final concentration of DMSO in the medium was less than 0.1% to avoid affecting cell viability.

### Experiment group

The HMrSV5 cells in the logarithmic growth phase were seeded in 24-well culture plates at a density of 5 × 10^5^ cells per well, in 500 μL of DMEM/F12 medium for incubation. Near-confluent cells were incubated with DMEM/F12 medium (200 μL) containing 0.5%FBS for 24 h to induce cell synchronization. Afterward, the medium was not replaced and cells were divided into the following groups: ① Control group: Cells were stimulated with an additional 200 μL of DMEM/F12 medium containing 0.5% FBS; ② PDS group: Cells were stimulated with 1.5% Dianeal, 2.5% Dianeal and 4.25% Dianeal 200 μL respectively; ③ Curcumin group: Cells were stimulated with 4.25% Dianeal (200 μL) and different concentrations of curcumin (20, 40 and 80 μmol/L). At 0, 12, 24, and 48 h, cells and culture supernatant were collected. The cells were centrifuged at 4 °C for 10 min to obtain supernatant, and stored at − 20 °C for testing.

### Cell viability assay

Cells were seeded in a 96-well plate with triplicates (5000 cells/well, 100 μL), and then incubated at 37 °C in a 5% CO_2_ incubator for 24 h. Afterward, the medium was not discarded and various treatments (100 μL) were applied. At selected time points (0,12,24,48,and72 h), CCK-8 solution (10 μL) was added to each well and incubated for 2 h at 37 °C. The absorbance at 450 nm was determined using a spectrophotometer.

### Transwell migration assay

In order to remove the influence of serum on the experiment, peritoneal mesothelial cells were treated with serum-free medium for 12 h, and then were resuspended in DMEM/F12 medium containing 1.0% FBS, and the cell density was adjusted to 5 × 10 5 cells/well. The cell suspension (20 μL) was added to the upper chamber of a 24-well plate Transwell chamber (Corning, Inc.), with the lower chamber containing 500 μL of 10% FBS DMEM/F12 medium. 4.25%PDS and curcumin (20, 40, 80 μmol/L) or curcumin (80 μmol/L) alone was added to the upper chamber and incubated for 24 h. Then, the cells were fixed with 4% paraformaldehyde and washed three times with PBS. The cells that had not migrated on the membrane were removed with a cotton swab, and the cells on the membrane were stained with 1% crystal violet for 5 min, and these migration cells were counted under a microscope.

### ELISA assay

The TGF-β1 protein content in culture supernatant was determined by ELISA kit according to the manufacturer’s instructions. After the coloration, the absorbance (A) value was read at a 450 nm wavelength to establish a standard curve, and the actual TGF-β1 content was determined and expressed as ng/L.

### Quantitative real-time PCR

Total RNA was extracted by Trizol from each group and cDNA was obtained by reverse transcription. Real-time PCR primers for E-cadherin, α-SMA, collagen I, fibronectin, TGF-β1 and GAPDH were synthesized by Shanghai Invitrogen (Table 1). Reaction systems contained 2 × SYBR Premix Ex Taq II (Tli RNaseH Plus) 10 μL, 50 × ROX Reference Dye 0.4 μL, template cDNA 2 μL, upstream and downstream primers 0.4 μL, and deionized water 7.2 μL. Amplification conditions were set as follows: 95 °C pre-denaturation for 30 s, followed by 40 cycles of 95 °C degeneration for 5 s and 60 °C annealing for 30 s. The human GAPDH gene was used as an internal reference. ΔΔCt = (target gene-internal reference) CT value-(control group target gene-control internal reference) CT value; relative mRNA expression amount = 2-ΔΔCt × 100%.

**Table 1:** Primer sequences

Genes	Forward primer	Reverse primer
α-SMA (122 bp)	CTATGAGGGCTATGCCTTGCC	GCTCAGCAGTAGTAACGAAGGA
E-cadherin (109 bp)	ATTTTTCCCTCGACACCCGAT	TCCCAGGCGTAGACCAAGA
Collagen I (119 bp)	GTGCGATGACGTGATCTGTG	CGGTGGTTTCTTGGTCGGT
Fibronectin (130 bp)	CGGTGGCTGTCAGTCAAAG	AAACCTCGGCTTCCTCCATAA
TGF-β1 (209 bp)	CTAATGGTGGAAACCCACAACG	TATCGCCAGGAATTGTTGCTG
GAPDH (282 bp)	GAGATCCCTCCAAAATCAAGTG	GAGTCCTTCCACGATACCAAAG

### Western blotting

Cells were rinsed with ice-cold PBS and lysed using RIPA buffer (150 mM NaCl, 1% charge, 0.5% sodium deoxycholate, 0.1% SDS, 50 mM Tris Cl, pH 7.4) containing 20% (v/v) cocktail of protease inhibitors (Sigma Aldrich). The lysates were centrifuged at 12,000×g at 4 °C for 10 min, and the supernatants were collected to measure protein concentration by the BCA Protein Assay Kit (Thermo, USA). Equal amounts of proteins (50 μg) were separated by 12% SDS-PAGE gel electrophoresis and then electrophoretically transferred to a PVDF membrane (Millipore, Bedford, USA). Membranes were incubated with 5% skim milk in TBST at 4 °C and then incubated with primary antibodies against human α-SMA, E-cadherin, p-TAK1, p-JNK and p- p38 (all 1:1000 dilutions) at 4 °C overnight. The membrane was then incubated with horseradish peroxidase-labeled secondary antibody (IgG) (11,000 dilution) for 1 h at room temperature. Band densities were visualized using a chemiluminescent detection system (ECL, Amersham Life Sciences, Buckinghamshire, UK) and Bio-Rad chemidoc XRS (Bio-Rad, USA). The protein band densities were converted into gray values, and relative expression was expressed as the target protein gray value normalized to β-actin.

### Statistical analysis

Data are expressed as means ± standard error of the mean (SEM) from at least three independent experiments, and analyzed by SPSS 19 statistical software. Differences between treatment groups were analyzed by t-test or analysis of variance (ANOVA), followed by the student Newman-Keuls (SNK) test. A two-tailed *P* value < 0.05 was considered to indicate statistical significance.

## Results

### Curcumin reversed viability inhibition of HPMCs under high glucose

**Fig. 1:**
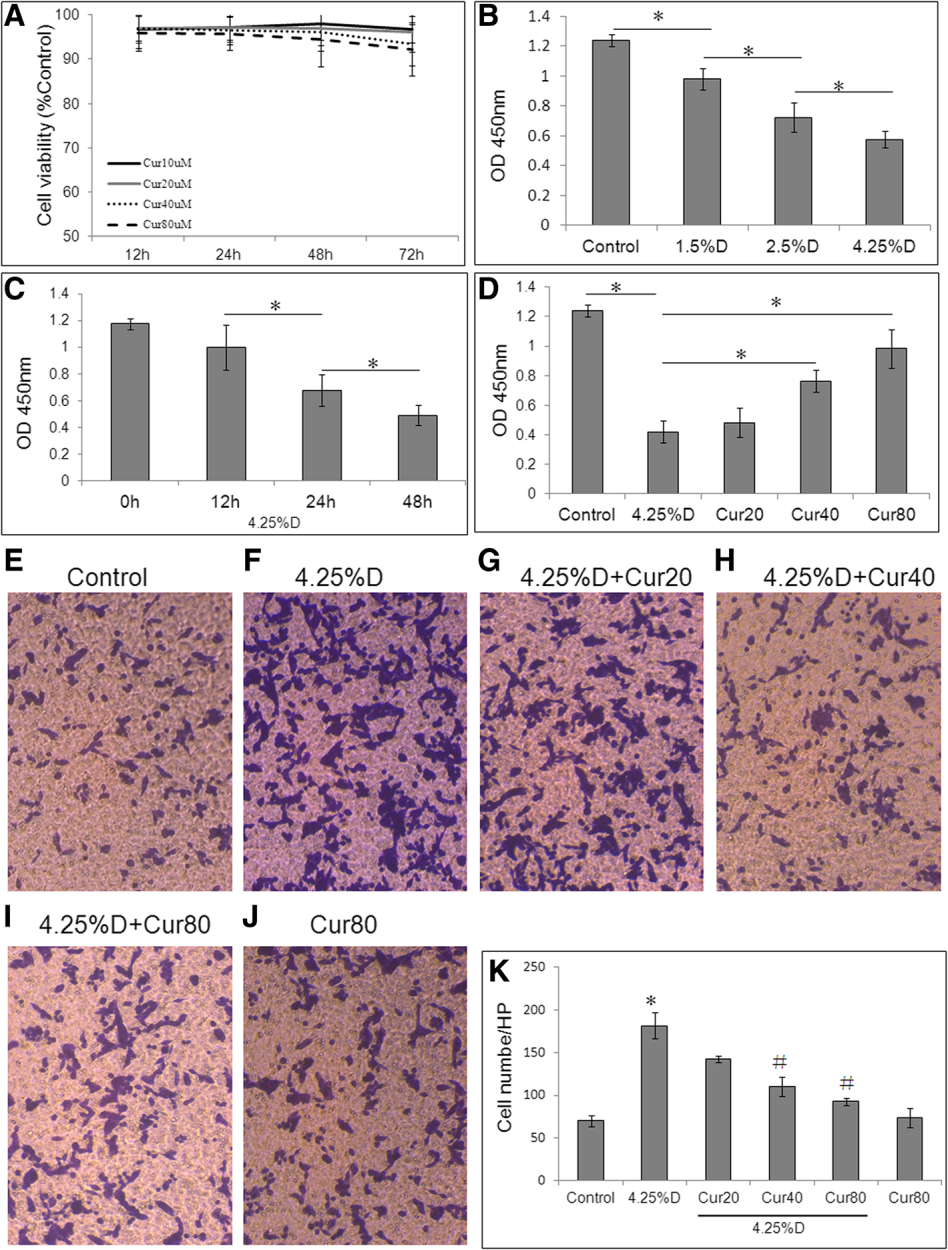
Effect of curcumin on HMrSV5 cell viability and migration. Cells were treated with various concentrations of curcumin (10, 20, 40 and 80 μM) for 12, 24, 48 and 72 h (**a**) or various concentrations of PDS (1.5% Dianeal, 2.5% Dianeal, 4.25% Dianeal) for 24 h (**b**) or PDS (4.25% Dianeal) for various times (0, 12, 24 and 48 h) (**c**). Cells were divided into control group (cells treated with DMEM/F12 medium containing 0.5%FBS), 4.25% Dianeal group, and 4.25% Dianeal + curcumin group (cells treated with 4.25% Dianeal and curcumin of 20, 40, and 80 μM) (**d**). Cell viability was measured by CKK-8 assay. *P < 0.05. Effect of curcumin on HMrSV5 cell migration was determined by Transwell assay, and the migrating cells were detected by crystal violet staining (**e**: control group; **f**: 4.25% Dianeal group; **g**: 4.25% Dianeal + curcumin 20 μM; **h**: 4.25%D + curcumin 40 μM; **i**: 4.25% Dianeal + curcumin 80 μM; **j**: curcumin 80 μM). Six random fields for each slice were counted, *n* = 3. *P < 0.05 vs. control group; #*P* < 0.05 vs. 4.25% Dianeal group **k**: The quantification of the migrating cells is expressed graphically

In order to select the appropriate curcumin intervention dose, we conducted a cytotoxicity experiment in HMrSV5 cells. Cells were treated with various concentrations of curcumin (10, 20, 40 and 80 μM) for different time points (12 h, 24 h, 48 h and 72 h). According to the CCK-8 assay, cell viability was not significantly decreased after 10, 20, and 40 μmol/L curcumin treatment at each time point, and was only slightly decreased after 80 μM curcumin treatment for 72 h (Fig. 1a). Then cells were treated with PDS (1.5%Dianeal, 2.5% Dianeal and 4.25% Dianeal) for 24 h, and it was found that PDS significantly decreased the viability of HMrSV5 cells in a concentration-dependent manner (*P* < 0.05) (Fig. 1b). We chose 4.25% Dianeal for further experiments and cells wwer incubated with 4.25% Dianeal for 0, 12, 24 and 48 h, respectively. The results showed that PDS significantly inhibited the proliferation of HMrSV5 cells in a time-dependent manner (*P* < 0.05) (Fig. 1c). To investigate the effect of curcumin on cell viability, cells were treated with 4.25% Dianeal plus various concentrations of curcumin (20, 40 and 80 μM) for 48 h co-culture. The results showed that curcumin at 40 and 80 μM could significantly reverse the decreased viability of HMrSV5 cells induced by PDS (*P* < 0.05) (Fig. 1d).

### Curcumin inhibits HPMC migration enhanced by high glucose

We also examined the inhibitory effect of curcumin on the cell migratory activity because cells undergoing EMT acquire higher mobility. Changes in cell migration were assessed using transwell assay. As shown in Fig. 1e-k, in the transwell assay, treatment with 4.25% Dianeal significantly increased the number of migrated HMrSV5 cells compared with the control (*P* < 0.05). Cotreatment with curcumin at 40 μM and 80 μM significantly inhibits PDS-stimulated cell migrating activity. Treatment with curcumin alone had no significant change compared with the control. Taken together, these findings suggested that high glucose PDS obviously induced EMT, and curcumin effectively inhibited the EMT progress.

### Curcumin attenuated glucose-induced EMT of HPMCs

**Fig. 2:**
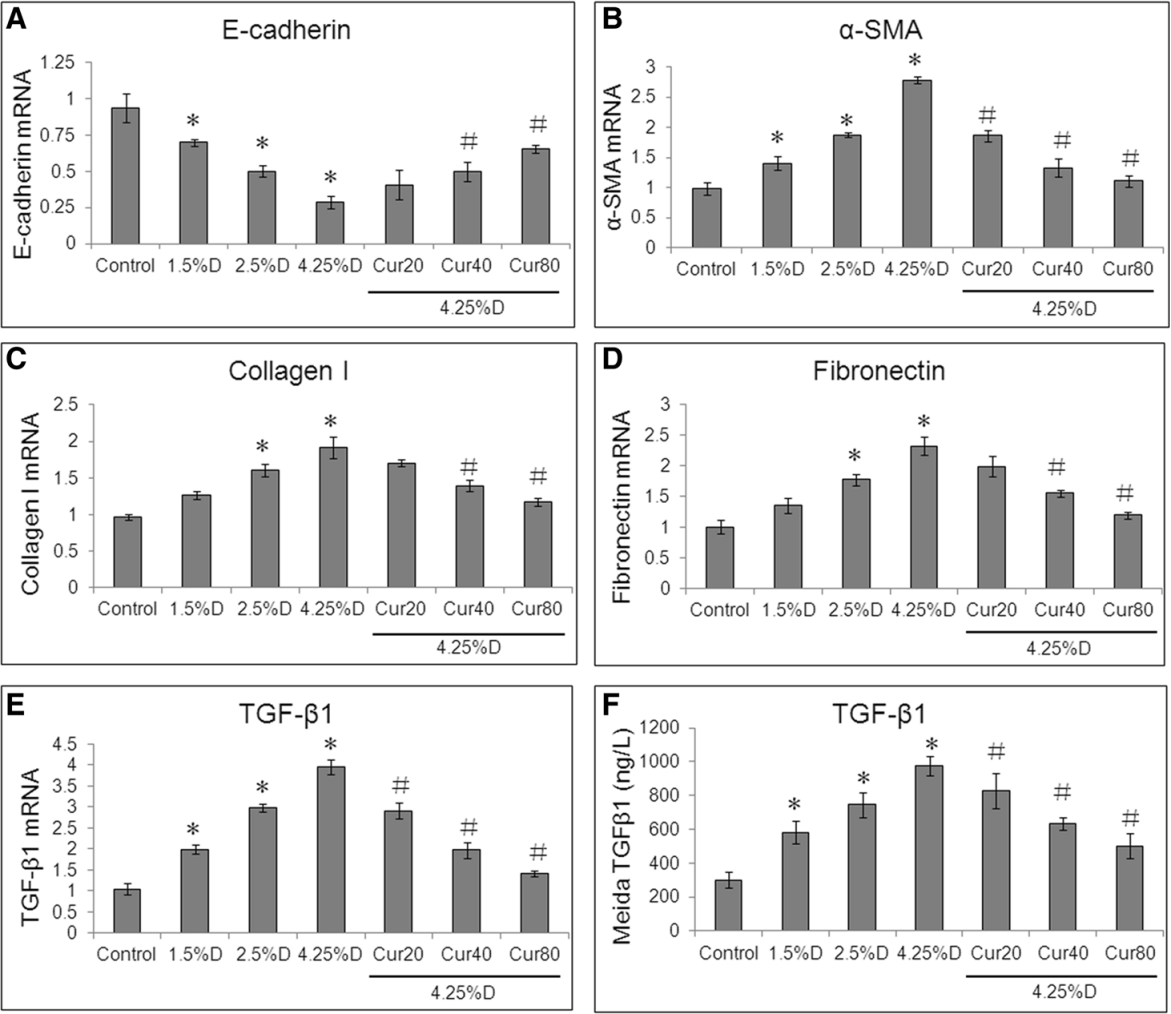
Effect of curcumin on PD-induced mRNA expressions of EMT-related genes in HMrSV5 cells. Cells were divided into control group (cells treated with DMEM/F12 medium containing 0.5%FBS), three PD groups (cells treated with 1.5% Dianeal, 2.5% Dianeal and 4.25% Dianeal), and three 4.25% Dianeal + curcumin group (cells treated with 4.25% Dianeal plus curcumin of 20, 40 and 80 μM). After incubation for 48 h, cells were lysed using TRIzol reagent for quantitative real-time PCR assay, and culture supernatant was collected for ELISA assay. GAPDH served as a loading control. Relative mRNA expressions of E-cadherin (**a**), α⁃SMA (**b**), collagen I (**c**), fibronectin (**d**) and TGF⁃β1 (**e**) are shown. TGF-β1 protein content in culture supernatant was determined (**f**). *P < 0.05 vs. control group; #*P* < 0.05 vs. 4.25% Dianeal group

Real-time RT-PCR showed that high glucose could induce EMT in peritoneal mesothelial cells. After treatment with 4.25% Dianeal for 48 h, the mRNA expression of epithelial marker E-cadherin was down-regulated (Fig. 2a), and the mRNA expressions of mesenchymal markers α-SMA (Fig. 2b), collagen I (Fig. 2c) and fibronectin (Fig. 2d) was up-regulated (All *P* < 0.05). Treatment with curcumin attenuated the down-regulation of E-cadherin and the up-regulation of α-SMA, collagen I and fibronectin in HMrSV5 cells (All P < 0.05). Furthermore, treatment with 4.25% Dianeal also significantly increased TGF-β1 mRNA expression (Fig. 2e) and supernatant TGF-β1 and collagen I protein content (Fig. 2f) (Both P < 0.05).

**Fig. 3:**
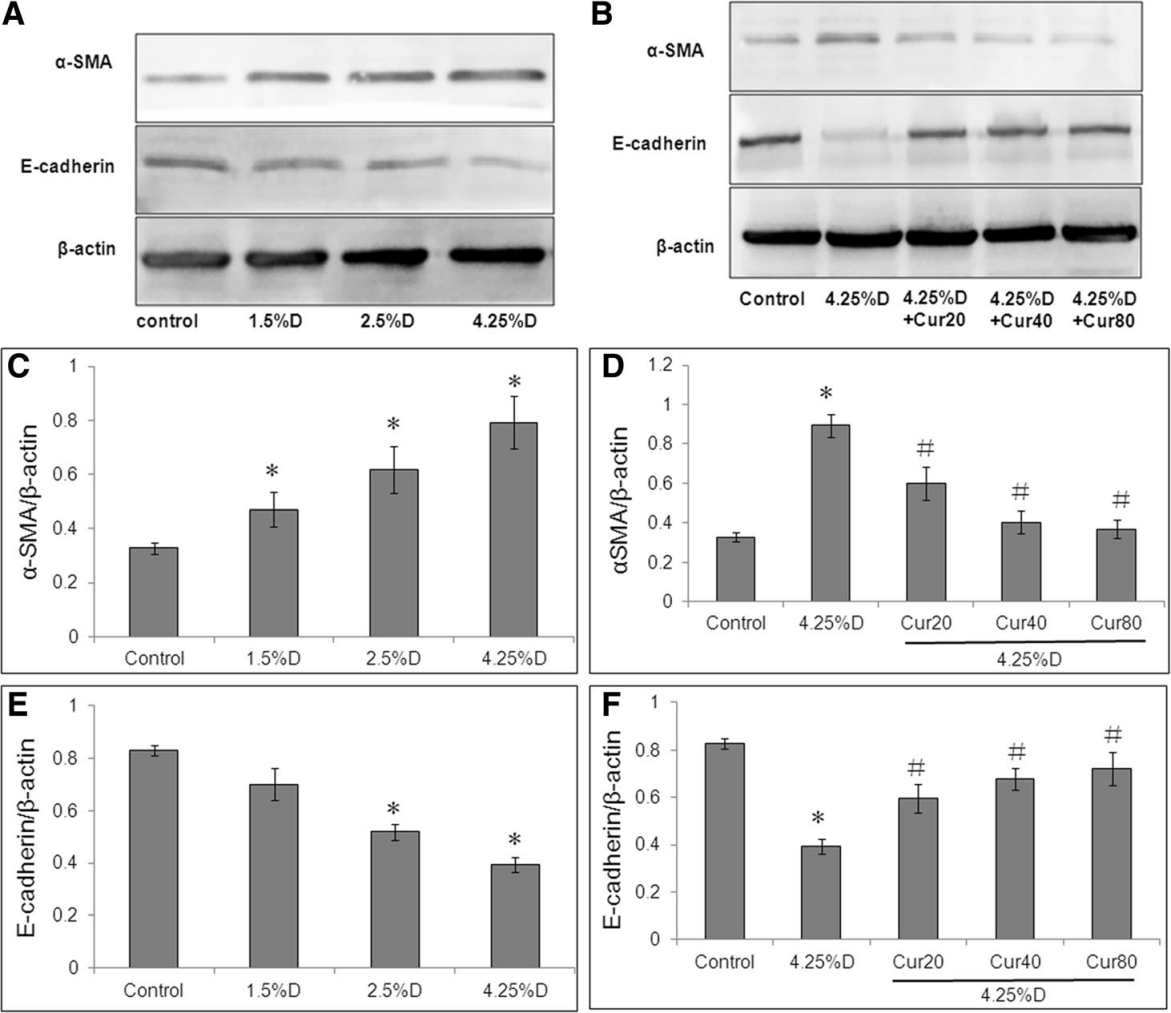
Effect of curcumin on PD-induced EMT marker protein expressions in HMrSV5 cells. Cells were divided into control group (cells treated with DMEM/F12 medium containing 0.5%FBS), three PD groups (cells treated with 1.5% Dianeal, 2.5% Dianeal and 4.25% Dianeal) and three 4.25% Dianeal + curcumin group (cells treated with 4.25% Dianeal plus curcumin of 20, 40 and 80 μM). After incubation for 48 h, cells were lysed using RIPA buffer for western blot assay. Representative immunoblots of α-SMA, E-cadherin and β-actin in HMrSV5 cells under various treatments are shown (**a**, **b**). β-actin served as a loading control. The density of protein bands were converted into grayscale values and are expressed as mean ± SEM. Relative protein expressions of α-SMA (**c**, **d**) and E-cadherin (**e**, **f**) are normalized to those of β-actin in HMrSV5 cells under various treatments. *P < 0.05 vs. control group; #*P* < 0.05 vs. 4.25% Dianeal group

Western blot analysis showed that high glucose could induce EMT in peritoneal mesothelial cells, as evidenced by representative images of decreased E-cadherin protein and increased α-SMA protein in HMrSV5 cells (Fig. 3a, b). The protein expression of mesenchymal marker α-SMA was increased by 4.25% Dianeal in a concentration-dependent manner, and was significantly decreased by curcumin (Fig. 3c, d). Meanwhile, the protein expression of the epithelial marker E-cadherin was decreased by 4.25% Dianeal in a concentration-dependent manner, and was significantly increased by curcumin (Fig. 3e, f).

### Curcumin attenuated EMT by activating TAK1 signaling pathways

**Fig. 4:**
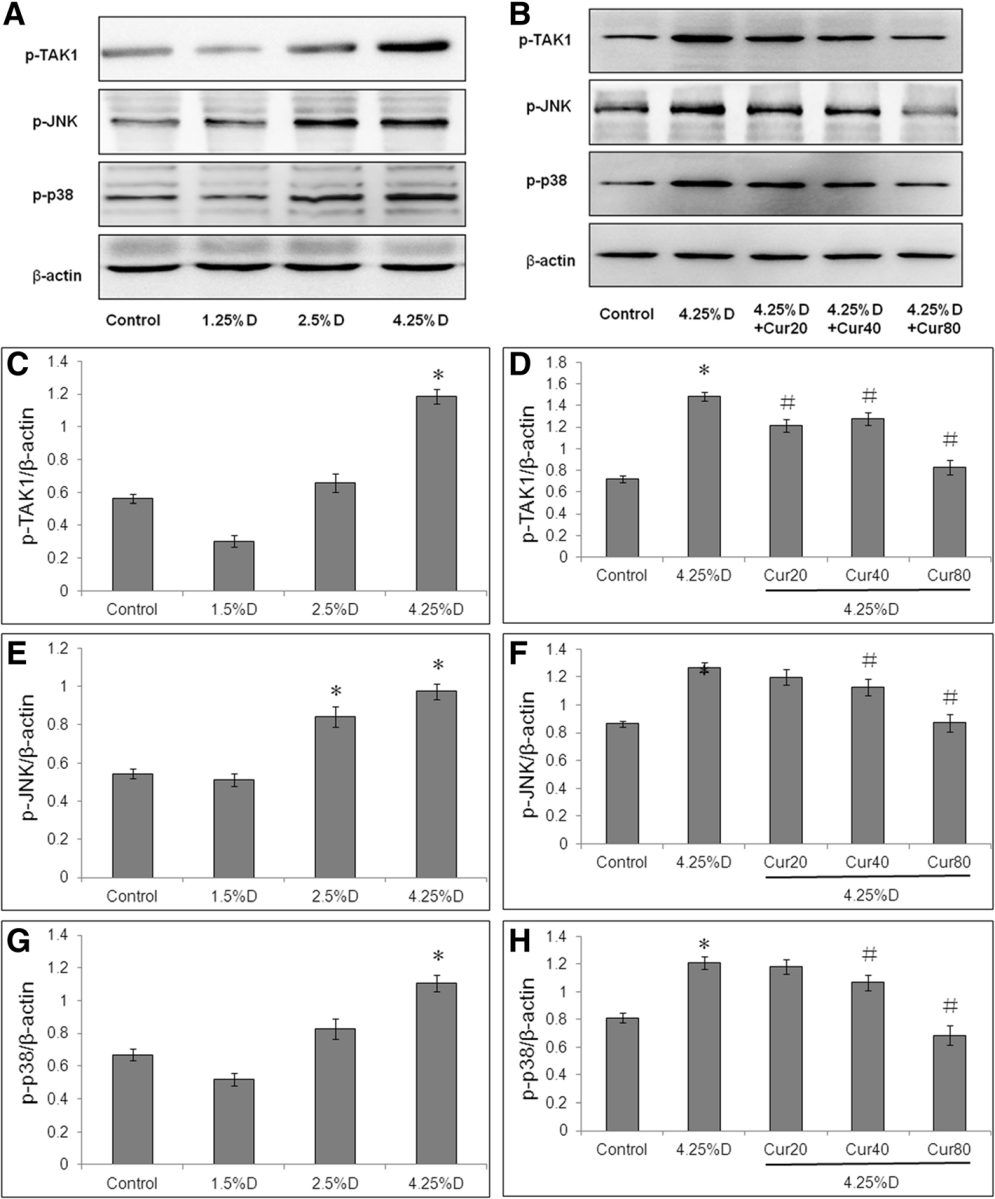
Effect of curcumin on PD-induced p-TAK1, p-JNK and p-p38 activation in HMrSV5 cells. Cells were divided into control group (cells treated with DMEM/F12 medium containing 0.5%FBS), three PD groups (cells treated with 1.5% Dianeal, 2.5% Dianeal and 4.25% Dianeal) and three 4.25% Dianeal + curcumin group (cells treated with 4.25% Dianeal plus curcumin of 20, 40 and 80 μM). After incubation for 24 h, cells were lysed using RIPA buffer for western blot assay, respectively. Representative immunoblots of p-TAK1, p-JNK, p-p38 and β-actin in HMrSV5 cells under various treatments is shown in (**a**, **b**). β-actin served as a loading control. After normalizing to protein bands grayscale of β-actin, relative intensity of p-TAK1 (**c**, **d**), p-JNK (**e**, **f**) and p-p38 (**g**, **h**) are shown in HMrSV5 cells under various treatments. *P < 0.05 vs. control group, #*P* < 0.05 vs. 4.25% Dianeal group

In order to explore the mechanisms underlying suppression of EMT by curcumin in glucose-induced HPMCs, western blot was performed to measure the protein levels of p-TAK1, p-JNK and p-p38. High glucose activated TAK1/JNK and TAK1/P38 pathways in a concentration-dependent manner during the induction of EMT in HMrSV5 cells, as shown in representative images (Fig. 4a, b). p-TAK1 protein level was significantly increased by 4.25% Dianeal treatment, and curcumin decreased p-TAK1 protein level in a concentration-dependent manner, with significant difference at higher concentrations (40, 80 μM) (*P* < 0.05) (Fig. 4c, d). Curcumin also significantly decreased protein levels of p-JNK and p-p38 (P < 0.05) (Fig. 4e-h). All the above results suggest an implication of TAK1, p38 and JNK pathway in mediating the inhibitory effect of curcumin in EMT of MCs.

## Discussion

Peritoneal fibrosis remains a serious complication of long-term PD leading to peritoneal membrane ultrafiltration failure. In the past few years, it has been identified that EMT of mesothelial cells (MCs) is an early and crucial process in the onset and progression of PD-related peritoneal fibrosis. Unfortunately, no effective methods to block EMT of MCs have been approved in clinical practice. Most studies thus far have focused on Chinese medicine materials as an alternative treatment. Curcumin is a polyphenol derived from turmeric. Both in vitro and in vivo experiments confirmed that curcumin shows anti-fibrotic effects on organ fibrosis [11–14, 18]. However, the potential effect and exact molecular mechanism of curcumin on PD-induced EMT of human peritoneal mesothelial cells have not been clearly elucidated.

In this study, we first assessed the role of curcumin during high glucose PDS-induced EMT in HMrSV5 cells. High glucose PDS decreased cell viability and increased migratory capacity, and curcumin increased cell viability and reduced migration capability of HPMCs. In HMrSV5 cells, high glucose PDS also decreased expression of epithelial markers, and increased expression of mesenchymal markers, a characteristic of EMT. Real-time RT-PCR and western blot revealed that, compared to the 4.25% Dianeal treated cells, curcumin treatment resulted in increased expression of E-cadherin (epithelial marker), and decreased expression of α-SMA (mesenchymal markers). Curcumin also reduced TGF-β1 mRNA and supernatant TGF-β1 protein content in the 4.25% Dianeal treated HMrSV5 cells. Furthermore, curcumin treatment also decreased protein expression of p-TAK1, p-JNK and p-p38 (downstream of TGF-β1). Recently, limited studies have shown the effect of curcumin in preventing EMT and peritoneal fibrosis in vivo and in vitro by inhibiting activating protein-1 (AP-1, [19]). In this study, we demonstrated that curcumin had an obvious effect in inhibiting PDS-related EMT of HMrSV5 cells and expression of TGF-β1. In addition, our results suggest an implication of the TAK1, p38 and JNK pathway in mediating the inhibitory effect of curcumin in EMT of MCs.

PMCs constitute the main cell population of the peritoneum and maintain its integrity, and participate in the local defense of the abdominal cavity. In the past, interstitial fibroblasts and inflammatory cells were considered to be the main cells responsible for PF, and PMCs were only passive victims of peritoneal injury. However, recent studies have found that PMCs are also active participants of PF, and myofibroblasts that transdifferentiated from PMCs play an important role in the occurrence of PF [20]. Therefore, we chose human peritoneal mesothelial cells (HMrSV5) to evaluate the in vitro effect of curcumin, and established a high glucose-induced PMCs model of EMT by incubating with 4.25% glucose peritoneal dialysis solution, which could adequately imitate the condition of PD [21]. Our study showed that high glucose-based PDS (1.5, 2.5, 4.25%) treated cells demonstrate reduced viability in a dosage-and time-dependent manner, which is in accordance with other reports. The reduced viability may be caused by high glucose-induced apoptosis and reactive oxygen species (ROS) production in HPMCs [22]. Our study showed that curcumin attenuated 4.25% Dianeal induced declined viability of HMrSV5 cells which indicates protective effects of curcumin against pathological process of HPMCs. EMT of PMCs is an early and crucial mechanism in the onset and progression of PD-related peritoneal fibrosis. Emerging evidence showed that methods to inhibit EMT could suppress peritoneal fibrosis and therefore preserve the peritoneal membrane. There are several genes implicated in EMT, including the repression of epithelial markers such as E-cadherin and cytokeratins, together with induction of mesenchymal markers such as α-SMA, vimentin, fibronectin and collagen I [23]. We specifically analyzed the expression of E-cadherin and α-SMA in mRNA and protein level as well as fibronectin and collagen I mRNA expression, which are the representative markers implicated in EMT. Our results showed that high glucose-based PDS (1.5, 2.5, 4.25%) effectively induced EMT in a dosage-dependent manner. Curcumin showed a remarkable protective effect on PDS-induced EMT of HMrSV5 cell, as it increased expression of E-cadherin and decreased a-SMA, fibronectin, and collagen I expressions. The acquisition of migratory capacity is another typical characteristic of cells undergoing EMT [5]. Therefore, we assessed the HMrSV5 cells migration by transwell assay. The results showed significant suppression of curcumin on increasing migration induced by high glucose-based PDS. Studies have found that TGF-β1 is the most important cytokine that induces the occurrence of EMT in PMCs. Long-term PD treatment exposes PMCs to high glucose, high glucose metabolites, high osmotic pressure, low pH dialysate and peritonitis, thereby stimulating the production of TGF-β1. TGF-β1 binds to TGF-β RI/II (TGF-β receptor) on the cell surface, activates intracellular signal transduction, and modulate expression of different genes, thus ultimately inducing EMT in PMCs [24]. Our results are in accordance with the observation that TGF-β1 mRNA and protein content in supernatant were increased by 4.25% Dianeal and significantly down-regulated by curcumin. This suggests that curcumin might inhibit peritoneal fibrosis partly through inhibiting TGF-β1-induced EMT of peritoneal mesothelial cells. Curcumin was proved to ameliorate TGF-β1-induced EMT in the suppression of renal fibrosis [25] and cardiac fibrosis [26].

TGF-β1-activated kinase1(TAK1) has emerged as a critical signaling molecule in TGF-β1-induced Smad-independent signaling pathways. After activation by TGF-β1, TAK1 can activate JNK and p38, respectively, thus regulating the transcription of target genes [27]. Recently, the TGF-β1/TAK1 pathway has been identified as an important participant process of TGF-β1-induced fibrosis. Inhibition of TAK1 suppressed EMT of primary human mesothelial cells [17] and inhibited peritoneal fibrosis of rats with long-term peritoneal dialysis [28]. These findings suggest that curcumin may suppress EMT-like changes through the inhibition of TAK1. To further investigate the downstream pathways of TGF-β1, we measured expression levels of TAK1, JNK and p38 in HMrSV5 cells by western blotting. The results showed that p-TAK1, p-JNK and p-p38 protein levels were significantly increased in HMrSV5 cells under high glucose PDS, which was significantly reduced following treatment with a middle and high dose of curcumin. There are only limited reports on modulation of TAK1 by curcumin. Curcumin attenuated hyperglycemia-mediated TAK1 protein expression in cerebrum of streptozotocin-induced diabetic rat [29]. Moreover, curcumin decreased the phosphorylation levels of TAK1 protein and p38 MAPK in mice with acute spinal cord injury [30]. The roles of TAK1 signal pathway in EMT and peritoneal fibrosis of PMCs are in accordance with a previous report [17]. Therefore, the suppression of TGF-β1-TAK1-JNK and TGF-β1-TAK1-p38 by curcumin will partly elucidate the exact molecular mechanism of peritoneal fibrosis.

In conclusion, the present study demonstrated that the TAK1-JNK and TAK1-p38 pathways are activated by high glucose in HPMCs and treatment with curcumin prevents EMT. This may implicate the TAK1, p38 and JNK pathway in mediating the inhibitory effect of curcumin in EMT of MCs. This study suggests that curcumin may be a good therapeutic agent for peritoneal fibrosis, and the role of TAK1 and its downstream pathway in EMT needs further research in peritoneal fibrosis.

## Conclusions

Our results showed that curcumin effectively suppresses EMT of glucose-induced HMrSV5 cells and suggest implication of the TAK1, p38 and JNK pathway in mediating the beneficial effects of curcumin. This indicates a potential therapeutic effect of curcumin on peritoneal fibrosis.

## Abbreviations

CCK-8: Cell Counting Kit-8
EMT: Epithelial–mesenchymal transition
ESRD: End-stage renal disease
GDPs: Glucose degradation products
HPMCs: Human peritoneal mesothelial cells
JNK: c-Jun N-terminal kinase
MCs: Mesothelial cells
PD: Peritoneal dialysis
PDS: Peritoneal dialysis solutions
PM: Peritoneal membrane
PMCs: Peritoneal mesothelial cells
TAK1: TGF-β-activated kinase 1
TGF-β1: Transforming growth factor-β1
UFF: Ultrafiltration failure
α-SMA: α smooth muscle actin
